# Supplementary material for: Risk factors associated with self-medication among the indigenous communities of Chittagong Hill Tracts, Bangladesh
Source: PLoS One. 2022 Jun 13;17(6):e0269622. doi: 10.1371/journal.pone.0269622 (PMC9191716; doi:10.1371/journal.pone.0269622)
Supplement: S1 Table — (DOCX) [file pone.0269622.s001.docx]

**S1 Table. Frequencies and chi-square test for socio-demographic variables and self-medication and types of self-medication practice**

| **Variable Group** | **General self- medication**  **(yes);**  **n= 1350 (%)** | | | | | **X^2^**  **(P-value)** | **Self-medication of analgesics & antipyretics**  **(yes);**  **n%** | **X^2^**  **(P-value)** | **Self-medication of anti-diarrheal & anti-ulcerants**  **(yes);**  **n%** | **X^2^**  **(P-value)** | **Self-medication of antibiotics**  **(yes);**  **n%** | | **X^2^**  **(P-value)** |
| --- | --- | --- | --- | --- | --- | --- | --- | --- | --- | --- | --- | --- | --- |
| **Age** | | | | | | | | | | | | | |
| 18-35 | 456 | | | | | **18.467**  **(0.001)** | 451 | **41.662**  **(0.001)** | 179 | **9.740**  **(0.136)** | 148 | | **0.376**  **(0.945)** |
| 36-50 | 126 | | | | |  | 152 |  | 58 |  | 50 | |  |
| 51-65 | 67 | | | | |  | 58 |  | 30 |  | 25 | |  |
| >65 | 25 | | | | |  | 26 |  | 8 |  | 10 | |  |
| **Gender** | | | | | | | | | | | | | |
| Male | 396 | | | | | **34.990**  **(0.001)** | 412 | **1.785**  **(0.410)** | 167 | **1.042**  **(0.594)** | 143 | | **12.396**  **(0.001)** |
| Female | 278 | | | | |  | 276 |  | 108 |  | 90 | |  |
| **Ethnicity** | | | | | | | | | | | | | |
| Chakma | | | | 319 | | **43.871**  **(0.001)** | 293 | **33.755**  **(0.001)** | 108 | **42.635**  **(0.001)** | 106 | | **12.390**  **(0.030)** |
| Marma | | | | 237 | |  | 268 |  | 108 |  | 81 | |  |
| Tripura | | | | 47 | |  | 38 |  | 18 |  | 19 | |  |
| Bawm | | | | 18 | |  | 27 |  | 14 |  | 3 | |  |
| Tanchangya | | | | 34 | |  | 41 |  | 15 |  | 15 | |  |
| Others* | | | | 19 | |  | 20 |  | 12 |  | 9 | |  |
| **Body mass index (BMI) kg/m^2^** | | | | | | | | | | | | | |
| <18.5 | | | | | 111 | **5.650**  **(0.059)** | 150 | **14.420**  **(0.006)** | 53 | **17.062**  **(0.000)** | | 36 | **4.438**  **(0.109)** |
| 18.5-23.5 | | | | | 368 |  | 372 |  | 145 |  |  | 123  74 |  |
| >23.5 | | | | | 195 |  | 165 |  | 77 |  |  |  |  |
| **Occupations** | | | | | | | | | | | | | |
| Agricultural work | | | 98 | | | **64.073**  **(0.001)** | 128 | **78.837**  **(0.001)** | 41 | **12.135**  **(0.145)** | 51 | | **14.373**  **(0.006)** |
| Service | | | 87 | | |  | 84 |  | 46 |  | 25 | |  |
| Housewife | | | 72 | | |  | 77 |  | 29 |  | 23 | |  |
| Student | | | 283 | | |  | 266 |  | 114 |  | 83 | |  |
| Others** | | | 128 | | |  | 132 |  | 45 |  | 51 | |  |
| **Educational Level** | | | | | | | | | | | | | |
| Illiterate | | 74 | | | | **39.947**  **(0.001)** | 83 | **77.554**  **(0.001)** | 28 | **7.288**  **(0.506)** | 28 | | **5.003**  **(0.287)** |
| Primary | | 77 | | | |  | 106 |  | 31 |  | 41 | |  |
| Secondary | | 106 | | | |  | 113 |  | 41 |  | 37 | |  |
| Higher secondary | | 187 | | | |  | 174 |  | 77 |  | 54 | |  |
| Graduate | | 230 | | | |  | 211 |  | 98 |  | 73 | |  |

***Other in ethnicity includes: Chak, Rakhine, Saotal and Mro**

****Others in occupation includes: Business, Day labor, Handloom and Unemployed**
