# Supplementary figures and images for: Risk factors associated with self-medication among the indigenous communities of Chittagong Hill Tracts, Bangladesh
Source: PLoS One. 2022 Jun 13;17(6):e0269622. doi: 10.1371/journal.pone.0269622 (PMC9191716; doi:10.1371/journal.pone.0269622)

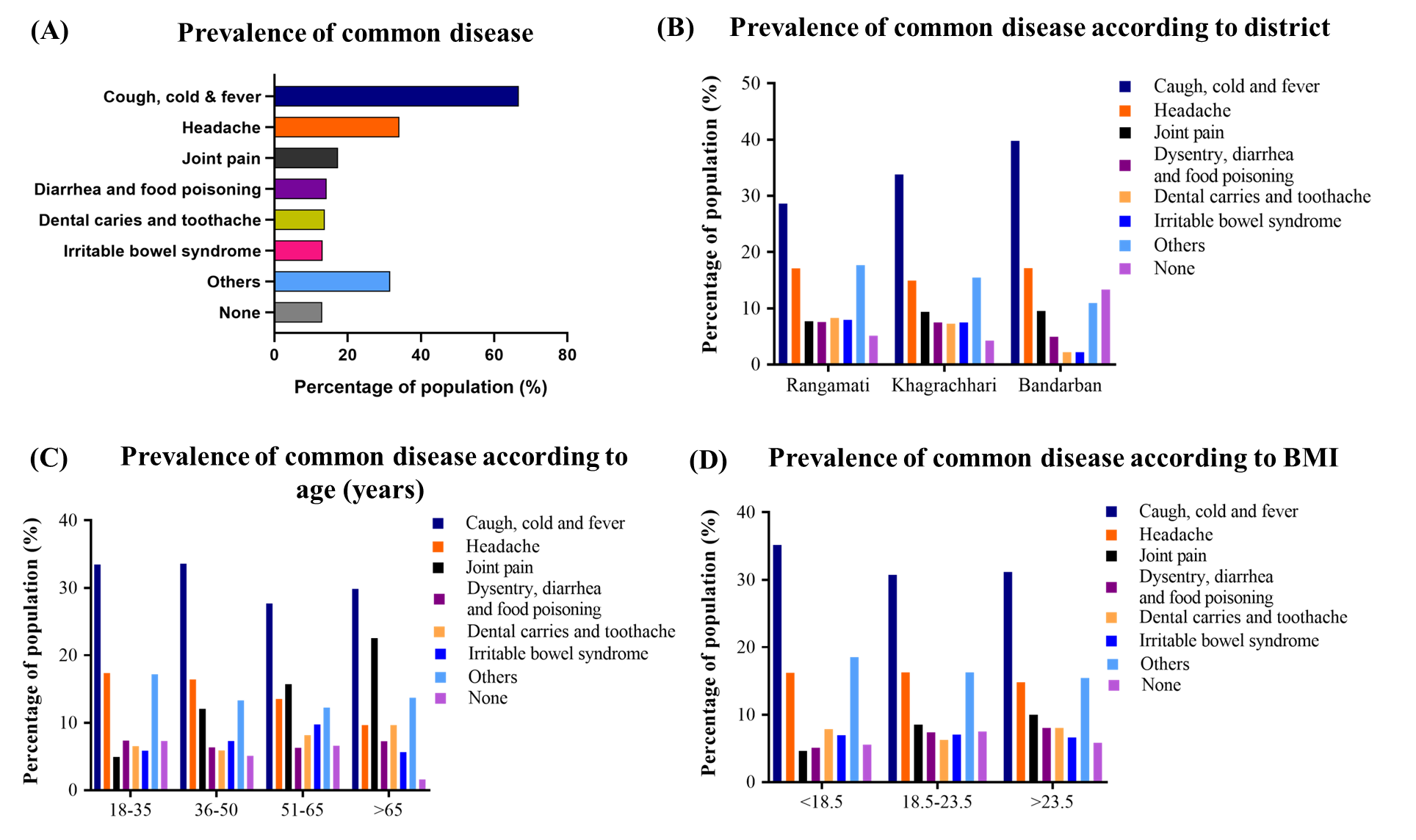

Supplement: S1 Fig — (A) Common disease prevalence among respondents; here Y-axis and the X-axis represent disease vs population percentage. (B) Common disease prevalence among districts, (C) disease prevalence among different age (years) groups and (D) disease prevalence among different BMI groups (<18.5 = underweight, 18.5–23.5 = normal weight, >23.5 = overweight); here Y-axis represents the percentage of the population and the X-axis represents districts, the age (years) of the respondents, and their BMI respectively. (TIF) [file pone.0269622.s002.tif]

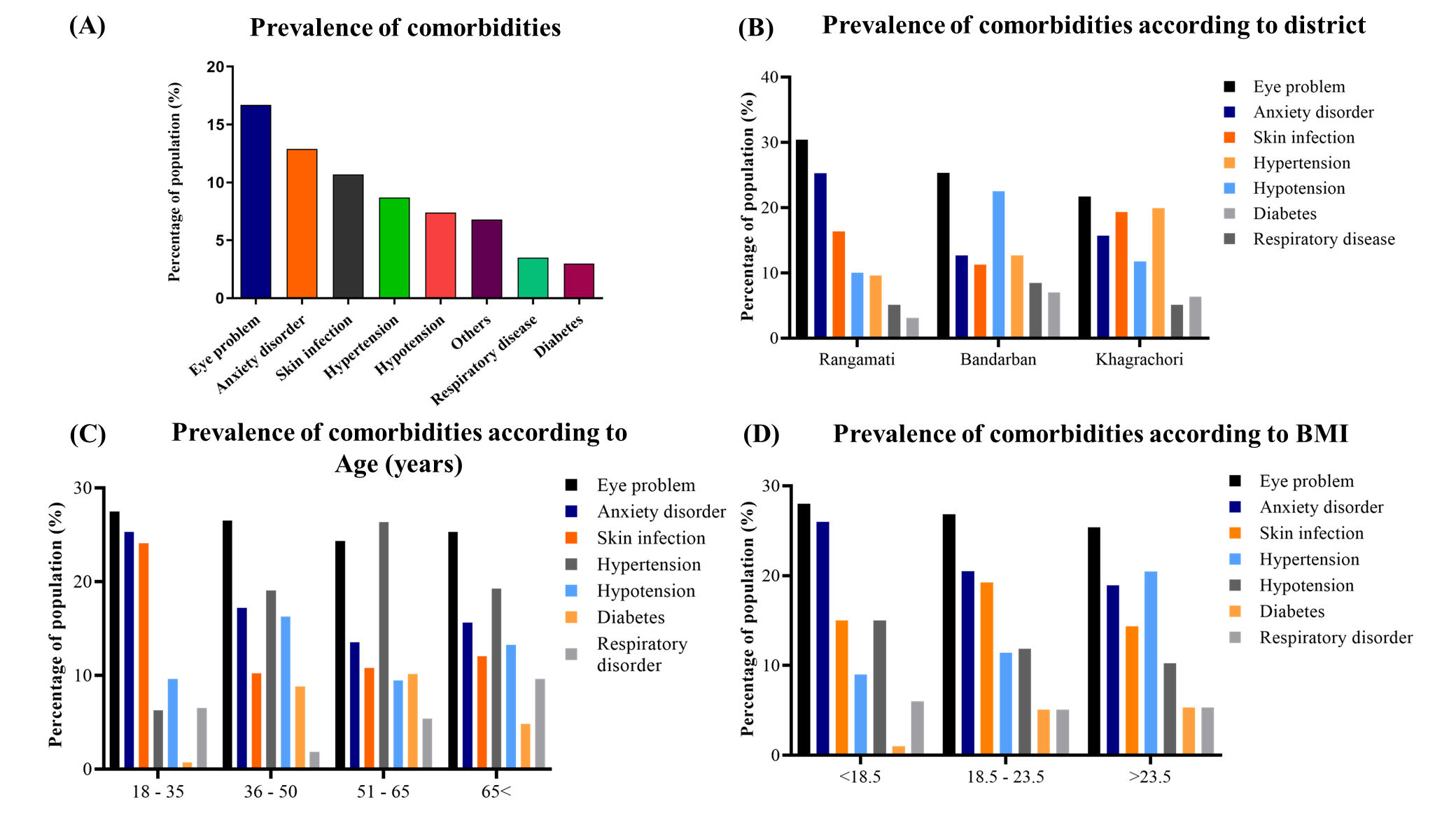

Supplement: S2 Fig — (A) prevalence of different types of comorbidities (B) prevalence of comorbidities based on districts (C) prevalence of comorbidities among different age (years) groups compared and (D) prevalence of comorbidities among different BMI groups (<18.5 = underweight, 18.5–23.5 = normal weight, >23.5 = overweight); here Y-axis indicates the percentage of population and X-axis indicates types of comorbidities, living districts, respondents age (years), and their BMI respectively. (TIF) [file pone.0269622.s003.tif]
