# Supplementary material for: Risk factors associated with self-medication among the indigenous communities of Chittagong Hill Tracts, Bangladesh
Source: PLoS One. 2022 Jun 13;17(6):e0269622. doi: 10.1371/journal.pone.0269622 (PMC9191716; doi:10.1371/journal.pone.0269622)
Supplement: S1 File — (PDF) [file pone.0269622.s005.pdf]

Self-medication among Indigenous People in Bangladesh

Interviewer's ID: | | | | | Date of interview:

The participant has given his/her consent to voluntarily participate in this study: | Yes | No |

1. Name:

2. Current address: Para/village: …………………..., Police station: ………….,

Post office: ………………. Upazila: …………………, District: …………………, Bangladesh

3. Permanent address: Para/village: …………………..., Police station: ………….,

Post office: ………………. Upazila: …………………, District: …………………, Bangladesh

4. Phone no (**Mandatory**):

➢ Questionnaire Section I: Socio Demographic Variables

5. Marital status: ☐ Never married ☐ Married ☐ Separated/Divorced ☐ Widow/widower

6. Age:

7. Ethnicity: ☐ Chakma ☐ Marma ☐ Tripura ☐ Tanchangya ☐ Bawm ☐ Others ……………

8. District: ☐ Khagrachhari ☐ Rangamati ☐ Bandarban

9. Height: ……… ft………. inch

10. Weight: ………….kg*

11. Gender: ☐ Male ☐ Female ☐ Others ……………

12. Educational Status:

☐ Illiterate ☐ Primary ☐ Secondary ☐ Higher secondary ☐ Graduate

13. Occupation:

☐ Agricultural work ☐ Day labor ☐ Business ☐ Healthcare work ☐ Handloom

☐ Housewife ☐ Service ☐ Student ☐ Unemployed ☐ Others …………….

- **Questionnaire Section II: Morbidities and medication practices**

14. Do you currently have any of the following diseases? (tick more than one if applicable)

☐ Heart disease	☐ Skin infection	☐Neurological disorder
☐ Respiratory disease	☐ Eye problem	☐ Kidney disease
☐ Hypertension	☐ Cancer	☐ Anxiety disorder
☐ Liver disease	☐ Diabetes	☐ Hypotension
☐ Thyroiditis	☐ None	☐ Not Applicable

15. Which of following diseases did you suffer in last one year? (tick more than one if applicable)

☐ Cough, cold & fever	☐ Headache
☐ Joint pain	☐ Diarrhea and food poisoning
☐ Asthma	☐ Dental carries & toothache
☐ Malaria	☐ Skin allergy
☐ Jaundice	☐ Irritable bowel syndrome
☐ Acne	☐ Roundworm/ Tapeworm
☐ Sinusitis	☐ Other respiratory illness
☐ Typhoid	☐ None
☐ Not applicable	

16. Have you ever taken medication due to any of the above mentioned (Q-18) conditions?

☐ Yes ☐ No

17. What kind of medicine did you take? (tick more than one if applicable)

☐ Antipyretics	☐ Analgesic	☐ Antibiotics	☐ Antacids and Anti-ulcerants
☐ Antidiarrheal	☐ Antitussive	☐ Anti-allergic	☐ Vitamins
☐ Antiemetic	☐ Sedatives	☐ Contraceptives	☐ Insulin
☐ Beta blockers	☐ Steroids	☐ Not applicable	

18. Source of that medicine (tick more than one if applicable):

□ Community pharmacy

□ Doctor's with prescription

□ Doctor's/ Community health workers without prescription

□ Self-medication experience from previous treatment

□ Previous prescription

□ Family & friends

□ Internet

□ Telemedicine

□ Folk medicine practitioner/ Kobiraj/ Ojha

□ Not applicable

19. How frequently did you use self-prescribed medication?

□ Never　□ Once in a year　□ 2-5 times in a year

□ 10 times/year　□ >10 times/year　□ Always

□ Not applicable

20. How far (in km) is the health care center from your residence?

Answer: …………. km

21. How far (in km) is the nearest pharmacy from your residence?

Answer: …..……. km

22. Monthly family income (in TK):

□ <20k　□ 20k - <35k　□ 35k -<50k

□ 50k - <75k　□ 75k - <100k　□ >100k
