# Supplementary material for: Risk factors associated with self-medication among the indigenous communities of Chittagong Hill Tracts, Bangladesh
Source: PLoS One. 2022 Jun 13;17(6):e0269622. doi: 10.1371/journal.pone.0269622 (PMC9191716; doi:10.1371/journal.pone.0269622)
Supplement: S2 File — (PDF) [file pone.0269622.s006.pdf]

বাংলাদেশে বসবাসরত আদিবাসী জনগোষ্ঠীদের মধ্যে স্ব-প্রণোদিত ঔষধ ব্যবহারের প্রবণতা

সাক্ষাৎকার গ্রহণকারীর আইডিঃ | | | | | সাক্ষাৎকার গ্রহণের তারিখঃ

সাক্ষাৎ প্রদানকারী স্বেচ্ছায় গবেষণায় অংশগ্রহণ করতে সম্মতি দিয়েছেন? | হ্যাঁ | না |

১. নাম :

২. বর্তমান ঠিকানা: পাড়া /মহল্লা /গ্রাম .... থানা .... ডাকঘর . . . .

উপজেলা . . . . জেলা . . . . , বাংলাদেশ।

৩. স্থায়ী ঠিকানা: পাড়া /মহল্লা /গ্রাম .... থানা .... ডাকঘর . . . .

উপজেলা . . . . জেলা . . . . , বাংলাদেশ।

৪. মোবাইল নাম্বার (আবশ্যক) :

- *১ম বিভাগ - সামাজিক জনসংখ্যা তথ্য :*

৫. বৈবাহিক অবস্থা : ☐ বিবাহিত ☐ অবিবাহিত ☐ ডিভোর্স ☐ বিধবা/ বিপত্নীক

৬. বয়স : .... বছর

৭. জাতিগোষ্ঠী : ☐ চাকমা ☐ মারমা ☐ ত্রিপুরা ☐ তঞ্চংগ্যা ☐ বম ☐ অন্যান্য . . . .

৮. বিভাগ : ☐ খাগড়াছড়ি ☐ রাংগামাটি ☐ বান্দরবান

৯. উচ্চতা : ....ফুট .... ইঞ্চি

১০. ওজন : .... কেজি

১১. লিঙ্গা : ☐ পুরুষ ☐ নারী ☐ অন্যান্য . . . .

১২. শিক্ষাগত যোগ্যতা : ☐ নিরক্ষর ☐ প্রাথমিক ☐ মাধ্যমিক ☐ উচ্চমাধ্যমিক ☐ স্নাতক

১৩. পেশা : ☐ কৃষিকাজ ☐ দিনমজুর ☐ ব্যাবসা ☐ চিকিৎসা কর্মী ☐ তাঁতশিল্প
☐ গৃহিণী ☐ চাকুরীজীবী ☐ ছাত্র ☐ বেকার ☐ অন্যান্য . . . .

- *২য় বিভাগ – শারীরিক অসুস্থতা ও ঔষধ গ্রহণের প্রবণতা বিষয়ক তথ্য :*

১৪. বর্তমানে আপনি নিম্নের কোন রোগে ভুগছেন? (প্রযোজ্য ক্ষেত্রে একাধিক টিক চিহ্ন দিন)

☐ হৃদরোগ ☐ চর্মরোগ ☐ স্নায়বিক রোগ ☐ শ্বাসপ্রশ্বাস জনিত রোগ ☐ চোখের সমস্যা

☐ কিডনির রোগ ☐ উচ্চরক্তচাপ ☐ ক্যান্সার ☐ অস্থিরতা ☐ লিভারের রোগ

☐ ডায়াবেটিস ☐ নিম্নরক্তচাপ ☐ থাইরয়েডের সমস্যা ☐ কোনোটিই নয় ☐ প্রযোজ্য নয়

১৫. গত এক বছরে নিম্নের কোন রোগে ভুগেছেন? (প্রযোজ্য ক্ষেত্রে একাধিক টিক চিহ্ন দিন)

☐ জ্বর/ সর্দি/ কাশি	☐ মাথাব্যথা	☐ বাতের ব্যাথা	☐ ডায়রিয়া
☐ হাঁপানি/ শ্বাসকষ্ট	☐ দাঁতের ব্যাথা / সমস্যা	☐ ম্যালেরিয়া	☐ চর্মরোগ
☐ জন্ডিস	☐ আইবিএস	☐ ব্রন	☐ কৃমিজনিত রোগ
☐ সাইনুসাইটিস	☐ অন্যান্য শ্বাসজনিত সমস্যা	☐ টাইফয়েড	☐ কোনটিই নয়
☐ প্রযোজ্য নয়			

১৬.উপরের রোগের কোনোটির (প্রশ্ন -১৫) জন্য ঔষধ খেয়েছিলেন? ☐ হ্যাঁ ☐ না

১৭. কোন ধরণের ঔষধ খেয়েছিলেন? (প্রযোজ্য ক্ষেত্রে একাধিক টিক চিহ্ন দিন)

☐ জ্বরের ঔষধ	☐ ব্যাথার ঔষধ	☐ এন্টিবায়োটিক	☐ গ্যাস্ট্রিকের ঔষধ
☐ ডায়ারিয়ার ঔষধ	☐ কফ নিবারক ঔষধ	☐ চুলাকানি/ এলার্জির ঔষধ	☐ ভিটামিন
☐ বমির ঔষধ	☐ ঘুমের ঔষধ	☐ জন্ম নিয়ন্ত্রন বড়ি	☐ ইনসুলিন
☐ হার্টের ঔষধ	☐স্টেরয়েড	☐ প্রজোজ্য নয়	

১৮. ঔষধের উৎস কোনটি ছিল (প্রযোজ্য ক্ষেত্রে একাধিক টিক চিহ্ন দিন)?

☐ স্থানীয় ঔষধের দোকান

☐ ডাক্তার (প্রেসক্রিপশনসহ)

☐ ডাক্তার/ কমিউনিটি চিকিৎসাকর্মী (প্রেসক্রিপশন ছাড়া)

☐ পুর্ব অভিজ্ঞতা

☐ পুর্বে প্রদানকৃত প্রেসক্রিপশন

☐ পরিবার অথবা বন্ধুবান্ধব

☐ ইন্টারনেট

☐ টেলিমেডিসিন

☐ ভেষজ/ কবিরাজি/ বৈদ্য চিকিৎসা

☐ প্রদানযোগ্য নয়

১৯. কতোবার ডাক্তারের পরামর্শ ছাড়া ঔষধ সেবন করেছেন?

☐ কখনো না	☐ বছরে এক বার	☐ বছরে ২-৫ বার
☐ বছরে ১০ বার	☐ বছরে ১০ বারের থেকে বেশি	☐ সবসময়
☐ প্রযোজ্য নয়		

২০. আপনার বাসা থেকে সবচেয়ে কাছের হাসপাতাল কতোদুরে (কিলোমিটার)?

উত্তর : .... কিলোমিটার

২১. আপনার বাসা থেকে সবচেয়ে কাছের ফার্মেসী কতোদুরে (কিলোমিটার)?

উত্তর : .... কিলোমিটার

২২. আপনার পরিবারের মাসিক আয় কতো (টাকায়)?

☐ <২০ হাজার ☐ ২০-<৩৫ হাজার ☐ ৩৫-<৫০ হাজার

☐ ৫০-<৭৫ হাজার ☐ ৭৫-<১ লাখ ☐ >১ লাখ
