# Supplementary material for: Risk factors associated with self-medication among the indigenous communities of Chittagong Hill Tracts, Bangladesh
Source: PLoS One. 2022 Jun 13;17(6):e0269622. doi: 10.1371/journal.pone.0269622 (PMC9191716; doi:10.1371/journal.pone.0269622)
Supplement: Attachment — Submitted filename: Response to reviewers comments.docx [file pone.0269622.s007.docx]

| Reviewer | Comments | Response |
| --- | --- | --- |
| Journal requirements | 1. When submitting your revision, we need you to address these additional requirements.  Please ensure that your manuscript meets PLOS ONE's style requirements, including those for file naming. The PLOS ONE style templates can be found at  https://journals.plos.org/plosone/s/file?id=wjVg/PLOSOne_formatting_sample_main_body.pdf and  https://journals.plos.org/plosone/s/file?id=ba62/PLOSOne_formatting_sample_title_authors_affiliations.pdf | Thank you. The title and body of the manuscript have been modified in accordance with the PLOSONE guidelines. |
|  | 2. Please amend your current ethics statement to address the following concerns:  a) Did participants provide their written or verbal informed consent to participate in this study?  b) If consent was verbal, please explain i) why written consent was not obtained, ii) how you documented participant consent, and iii) whether the ethics committees/IRB approved this consent procedure. | a) Before participating in the study, participants provided verbal consent (Supporting Information file 1 and 2). It is mentioned in the section on Data Collection (Line 164).  b) i) During the data collection period, the Covid19 situation in Bangladesh was severe. We wanted to reduce any physical contact between participants and interviewers in order to ensure their safety. As a result, just verbal consent was obtained while maintaining a safe distance. ii) All the data were first manually recorded in paper (Supporting Information file 1 and 2) and then transferred to Google forms software to be exported and stored in Microsoft Excel 2013. |
|  | 3. In your Data Availability statement, you have not specified where the minimal data set underlying the results described in your manuscript can be found. PLOS defines a study's minimal data set as the underlying data used to reach the conclusions drawn in the manuscript and any additional data required to replicate the reported study findings in their entirety. All PLOS journals require that the minimal data set be made fully available. For more information about our data policy, please see http://journals.plos.org/plosone/s/data-availability.  Upon re-submitting your revised manuscript, please upload your study’s minimal underlying data set as either Supporting Information files or to a stable, public repository and include the relevant URLs, DOIs, or accession numbers within your revised cover letter. For a list of acceptable repositories, please see http://journals.plos.org/plosone/s/data-availability#loc-recommended-repositories. Any potentially identifying patient information must be fully anonymized.  Important: If there are ethical or legal restrictions to sharing your data publicly, please explain these restrictions in detail. Please see our guidelines for more information on what we consider unacceptable restrictions to publicly sharing data: http://journals.plos.org/plosone/s/data-availability#loc-unacceptable-data-access-restrictions. Note that it is not acceptable for the authors to be the sole named individuals responsible for ensuring data access.  We will update your Data Availability statement to reflect the information you provide in your cover letter. | 3. We have uploaded the data set (Supporting Information file 3) with revised manuscript for this study.  We don’t have any ethical or legal restrictions to sharing this data publicly. |
|  | 4. Please remove your figures and supplementary figures from within your manuscript file, leaving only the individual TIFF/EPS image files, uploaded separately. These will be automatically included in the reviewers’ PDF.  Please review your reference list to ensure that it is complete and correct. If you have cited papers that have been retracted, please include the rationale for doing so in the manuscript text, or remove these references and replace them with relevant current references. Any changes to the reference list should be mentioned in the rebuttal letter that accompanies your revised manuscript. If you need to cite a retracted article, indicate the article’s retracted status in the References list and also include | The manuscript's figures and supplementary figures have been removed and uploaded separately as TIFF image files.  All of the references have been double-checked.  The following references have been added to the revised copy: 33, 49, 51, 56, and 57. |
| Reviewer 1 | The study was conducted partly on PLOS one requirement. But my opinion is that it is a duplication of work done by other researchers. Nothing innovative in the methodology. But it can be approved by considering the fact that such studies are not done in the particular population | Thank you for your comment. We acknowledge your concept that this type study was done in Bangladesh but to the best of our knowledge this is the first study that has been carried out among the indigenous population in Bangladesh. So, in this context it adds novelty to the existing literature |
| Reviewer 2 | After thoroughly going through the research article, I present my comments below.  1) The authors in the results section, represented their finding in the form of overall Odds ratio and also Adjusted Odds ratio but did not mention the factors/aspects that were adjusted for. | 1) Thank you for bringing this to our attention. In the Statistical Analysis section (Line 173), we have gone through the details of calculating the adjusted and odd ratios. |
|  | 2) The authors in the study design section mentioned that they received insufficient responses from the participants. It would have been clearer for the readers if information regarding the insufficient response is mentioned and how the authors managed the data regarding this insufficient response. | 2) Thank you for bringing this to our knowledge. We clarified the participants response number in our manuscript and rewrite the sentence in line 120. |
|  | 3) Self medication practices followed by the sample population in this study includes both over the counter (OTC) medications and Prescription drugs. Combining both OTC medications and Prescription drugs to draw a conclusive outcome may be a concern for validity of the overall outcome of the study. | 3) We defined the self-medication as use of over-the-counter medications as well as the use of medications previously prescribed by doctor for a disease and taking the same medications for the current episode of that disease without consulting a doctor. We added this in the line 129 of the manuscript. |
|  | 4) It would have been a great help if the questionnaire used in the research was sent along with the manuscript. Lack of access to the questionnaire made it difficult to understand the factors that were considered in the analysis did make the process of reviewing the research article a bit difficult. | 4) We uploaded separate file of the questionnaire (Supporting Information file 1 and 2) as supporting file. |
| Reviewer 3 | The authors have represented the manuscript technically with satisfactory statistical analysis. Authors need to justify and elaborate the experimental findings in the discussion section more clearly. The impact (positive or negative) of education or literacy rate, economical and social standards of living on the self medication need to be discussed as the results have shown some odd findings (that need explanation) such as increase in the incidences of self medication in more educated and higher income people. In what type of problem (like social, economical, problems related to education, ethics, laws, etc.), the self medication should be included. What type of measures should be taken by the government and/ or health department? Any specific or even general preventive measures of self medication by the students as this group form the future of a country? | Thank you very much for sharing your ideas. In the revised manuscript, we have discussed how higher education and low income may influence self-medication practise among the indigenous population of the Chittagong hill region, as per your recommendation. We've cited references to back up our statements (Line 311, line 334). Our recommendations to policymakers are also mentioned in the discussion's final paragraph (Line 371). |
