# Supplementary material for: Risk factors associated with self-medication among the indigenous communities of Chittagong Hill Tracts, Bangladesh
Source: PLoS One. 2022 Jun 13;17(6):e0269622. doi: 10.1371/journal.pone.0269622 (PMC9191716; doi:10.1371/journal.pone.0269622)
Supplement: Attachment — Submitted filename: Response to reviewers comments_R2.docx [file pone.0269622.s008.docx]

**Dear Editor,**

We greatly appreciate the valuable comments from the reviewer and have modified the enclosed manuscript accordingly. Here, we include our responses to the reviewer’s comments.

| Reviewer 2 | With respect to the response given by the authors to the first comment, they mentioned the statistical method used for calculation of odds ratio and adjusted odds ratio. But what is actually asked for are the factors that were considered while adjusting the odds ratio. For example, what factors were considered for adjusted odds ratio of 0.60 in 18-35 year age group when compared to the crude odds ratio of 1.92. (Table 3) | Thanks for your comment. For your clarification the model building approach is presented in Methods section as follows:  “*The variables with a p value of less than 0.25 in the unadjusted analysis was included in the final multiple regression model.*” (Please see line 173-174).  We have also clarified this issue in more detail in the Result section as follows:  *“All the variables had a p value less than 0.25 in the bivariate analysis and thus all of them were included in the multivariable model. Hence, the model is adjusted for all the variables in Table 3.”* (Please see line 270-272). |
| --- | --- | --- |
