# Supplementary material for: Risk factors associated with self-medication among the indigenous communities of Chittagong Hill Tracts, Bangladesh
Source: PLoS One. 2022 Jun 13;17(6):e0269622. doi: 10.1371/journal.pone.0269622 (PMC9191716; doi:10.1371/journal.pone.0269622)
Supplement: Attachment — Submitted filename: Response to reviewers comments_R2.docx [file pone.0269622.s009.docx]

**Dear Editor,**

We greatly appreciate the valuable comments from the reviewer and have modified the enclosed manuscript accordingly. Here, we include our responses to the reviewer’s comments.

|  | Comment | Response |
| --- | --- | --- |
| Reviewer 2 | Thank you for the response for the comments provided. Now all the comments are addressed and explained. The authors mentioned that the variables whose p-value is less than 0.25 were considered in the final analysis of adjusted odds ratios. So all the parameters mentioned in table.2 were considered while deriving the adjusted odd's ratio mentioned in table 3? | Yes, we have considered all the socio-demographic characteristics of the participants presented in table 1. We performed bivariate analysis with each of them and those of p value less than 0.25 in the bivariate analysis were included in the multiple regression model. We have clarified this in the revised manuscript. Please see page 21 line 261. |
